# Addressing Conflicts of Interest and Conflicts of Commitment in Public Advocacy and Policy Making on CRISPR/Cas-Based Human Genome Editing

**DOI:** 10.3389/frma.2022.775336

**Published:** 2022-04-27

**Authors:** Alexander Christian

**Affiliations:** DCLPS, Institute of Philosophy, Heinrich-Heine-University, Duesseldorf, Germany

**Keywords:** conflict of interest, conflict of commitment, CRISPR/Cas, policy making, human genome editing

## Abstract

Leading experts on CRISPR/Cas-based genome editing—such as 2020 Nobel laureates Jennifer Doudna and Emmanuelle Charpentier—are not only renowned specialists in their fields, but also public advocates for upcoming regulatory frameworks on CRISPR/Cas. These frameworks will affect large portions of biomedical research on human genome editing. In advocating for particular ways of handling the risks and prospects of this technology, high-profile scientists not only serve as scientific experts, but also as moral advisers. The majority of them currently intend to bring about a “responsible pathway” toward human genome interventions in clinical therapy. Engaging in advocacy for such a pathway, they issue moral judgments on the risks and benefits of this new technology. They declare that there actually is a responsible pathway, they draft resolutions on temporary moratoria, they make judgments on which groups and individuals are credible and should participate in public and semi-public debates, so they also set the standards for deciding who counts as well-informed, as well as the standards of evidence for adopting or rejecting research policies. This degree of influence on public debates and policy making is, at the very least, noteworthy. This contribution sounds a note of caution with regard to the endeavor of a responsible pathway to human genome editing and in particular scrutinizes the legitimacy of expert-driven research policies given commercial conflicts of interest and conflicts of commitment among first-rank scholars.

## Introduction

The CRISPR/Cas technology has changed the landscape of biomedical research and genome engineering (Jinek et al., 2012; Hsu et al., 2014; Lin Y. et al., 2014). Due to its significant advantages over alternative technologies based on zinc-finger nuclease (ZFN) or TAL effector nuclease (TALEN), we now have access to more cost-effective, more precise, and more broadly applicable genome editing tools (Doudna and Charpentier, 2014; Ledford, 2015). Yet the prospects of human genome editing in controversial scenarios—in particular heritable editing—raises a series of complicated bioethical and legal ethical issues (Chan and Sternberg, 2019). The resolution of these issues has become an urgent matter in the wake of a research scandal surrounding the biophysicist He Jiankui in 2018.^1^ He was responsible for an experiment in which a CCR5-Δ32 mutation in human embryos was induced *via* CRISPR/Cas9 to bring about an immunity against HIV infections. It resulted in a renewed interest in an ongoing debate on the regulatory framework for future research on heritable human genome editing and pleas on a moratorium on human germline editing.^2^ Some experts have argued against a moratorium (Konig, 2019; Macintosh, 2019), whereas others have proposed risk-averse policies and endorsed a moratorium on clinical research, which could give policy makers and legislators time to establish international frameworks and develop ethical and legal guidelines on a national level (Lander et al., 2019). What complicates these debates is the fact that the CRISPR/Cas technology is a very economically valuable sector within the fast growing market of biotechnology (Brinegar et al., 2017). It is thus not surprising that many of the leading experts have ties to biomedical and pharmaceutical companies, e.g., receive funding for projects from pharmaceutical companies, have founded companies working with CRISPR/Cas themselves, own shares of biomedical companies or serve on scientific advisory boards.

My focus in this paper will be on conflicts of interest and conflicts of commitment in the context of public advocacy and public policy making on heritable human genome editing. According to a classical definition given by Thompson, a conflict of interest is “a set of conditions in which professional judgment concerning a primary interest (such as a patient's welfare or the validity of research) tends to be unduly influenced by a secondary interest (such as financial gain)” (Thompson, 1993, p. 375). Typically, conflicts of interest in biomedical research and medical practice emerge from financial ties between scientists and medical professionals and representatives of commercial entities like pharmaceutical companies. Yet it is important to note that every institutional system which works with financial or social incentives can produce conflicts of interest. For instance, if a professional agent has high expectations regarding her own work and is emotionally and motivationally dependent on positive feedback from professional peers, than the urge for acknowledgment by peers (secondary interest) can be in conflict with her professional obligations in research, like carefulness in conducting medical experiments (primary interest), when she rushed to publications in hope of acknowledgment. Often professional agents are not aware that they have conflicts of interest or act in a situation in which there is a more or less severe risk of biased decision-making (Bornstein and Emler, 2001; Felser and Klemperer, 2011, p. 29), which is why one of the most common strategies to cope with conflicts of interest is their declaration. This enables third parties to be aware of potential biases. While conflicts of interest, in particular the influence of commercial interests in biomedical research are widely discussed (cf. Lieb et al., 2011, 2018; Krimsky, 2018), the related concept of conflict of commitment receives less attention. Patricia Werhane and Jeffrey Doering define it as follows:

“*Conflicts of commitment are conflicts between at least two sets of professional obligations. Conflicts of commitment differ from conflicts of interest because conflicts of commitment involve the distribution of focus and effort between two sets of professional obligations, rather than a conflict between professional and financial/recognition interests. Conflicts of commitment are those conflicting commitments where competing obligations prevent honoring both commitments or honoring them both adequately.” (Werhane and Doering*, *1995**)*

Since conflicts of commitment emerge from professional obligations—and not from a conflict between primary interests (professional obligations) and secondary interests (like financial incentives and acknowledgment)—they are much harder to avoid on an institutional and individual level. One example for this is the commitment to contribute an equal or contractually defined distribution of time and attention to research, teaching, administrative duties, science communication, and public advocacy. Another example for conflicts of commitment is the conflict between prima facie legitimate research interests on the one hand and professional responsibilities in debates on research policies on the other, which affect the pursuit of those research interests. Think about a biomedical scientist who is committed to understand certain aspect of the development of human embryos, who also serves on an ethics committee which is tasked with the development of guidelines for human embryo research. Here, research interests in certain topics (a primary interest) could negatively affect the moral evaluation of the acceptability of experiments with human embryos (also a primary interest). In such a situation the researcher might favor self-serving guidelines, which enable the pursuit of certain research questions with regard to human embryos. Both types of conflicts create a risk for the moral integrity and objectivity of research and publications processes, efforts of science communication as well as policy making processes with regard to the CRISPR/Cas technology, as will be illustrated and discussed in later sections of this contribution.^3^ One particular problem in this context is that it is hard to distinguish between conflicts of commitment (resulting from conflicting professional obligations) and conflicts of interest (resulting from the presence of commercial interests), which is why both types of conflicts are addressed in this contribution and information is presented which allows for conclusions into the motivation for certain professional decisions.

I will argue that we need to establish stronger precautionary measures with regard to the disclosure of conflicts of interest and conflicts of commitments of leading experts at CRISPR/Cas-based genome editing by showing four things: First, information on commercial conflicts of interest of leading experts is sometimes not readily available and lacking in detail. Second, conflicts of interest usually are not disclosed in the context of public advocacy for specific research policies. This makes it very hard for participants in public discussions of ethical implications of the CRISPR/Cas technology to understand the actual economic interests in the background of certain advocated positions within the spectrum of risk-affirmative and risk-aversive positions. Third, the extent to which scientific experts on CRISPR/Cas are currently being relied on in public debates and policy making disregards philosophical insights into important differences between moral expertise and scientific expertise as well as deference to experts of either types. Fourth, the magnitude of influence experts on CRISPR/Cas have on public and semi-public debates as well as on policy making processes raises a political problem of legitimate representation. After making the case for increasing the transparency of public and semi-public debates with regard to conflicts of interest and conflicts of commitment, I will also shortly indicate the limitations of this approach for safeguarding the integrity of public debates and securing a responsible conduct with CRISPR/Cas.

This contribution is structured as follows: Section CRISPR/Cas9—What it is and what it does gives a brief and informal overview on the CRISPR/Cas technology in the context of human genome editing. Section An epic scientific misadventure familiarizes the reader with the research scandal surrounding the first attempt by He Jiankui to edit the genome of human embryos resulting in the birth of several children and summarizes critical reactions to this scandal from biomedical scientists and bioethicists. Section Experts in moral debates on the ethical issues of CRISPR/Cas technology and policy making first highlights two major consequences of this case with regard to the regulation of CRISPR/Cas-based human genome editing: the debate of a moratorium on heritable human genome editing and the work toward a responsible pathway. It then identifies the various ways in which scientific experts participate in public and semi-public ethical debates and in public policy making. Section A plea for caution then brings forward a series of philosophical concerns relating to this kind of involvement of biomedical experts and raises a note of caution with regard to the lack of transparency about commercial conflicts of interest and conflicts of commitment among experts in the CRISP/Cas technology. In the final section Toward more transparency, I discuss precautionary measures to safeguard the integrity and transparency of public and semi-public debates on ethical issues with the CRISPR/Cas technology.

## Crispr/Cas9—What it is and What it Does

The CRISPR/Cas technology is basically a toolkit for building molecular scissors (endonucleases), which can make genetic alterations at specifically chosen places in a DNA sequence (customized sequence specificity). CRISPR associated proteins (Cas) can be utilized for genome editing in various species (Jinek et al., 2012; Ran et al., 2013). One particular important type of endonuclease is Cas9. It has been shown that the CRISPR/Cas immune system of *Streptococcus pyogenes* can be used to create an active endonuclease complex consisting of Cas9 and guide RNA (gRNA/sgRNA). Due to the guide RNA, Cas9 can target specific genetic sequences and make genetic alterations, e.g., in human cells. With the help of this genetic tool, which was further developed into an entire toolkit for multiple purposes, scientists can comparatively easily target specific genetic sequences and make several types of changes (Makarova et al., 2015; Moon et al., 2019). Further details on the basics of CRISPR/Cas-based genome editing are explained inter alia in Yamamoto (2015), Gaj et al. (2016), and Luo (2019), I focus on the basics here.

It is important to note that the CRISPR/Cas technology has several advantages over alternative methods for genome editing, like ZFN and TALEN. For instance, nuclease design and assembly is easy and feasible in most labs, the success rate of nuclease design is high, the target specificity is high with most guide RNAs, the target range is potentially unlimited, multiplexing is highly feasible and it is not sensitive to CpG methylation (Gilles and Averof, 2014). This means that the CRISPR/Cas technology costs only a fraction of alternative methods, is faster and less labor intensive, it is very precise and it can be used to target a large range of genome sections. In biomedical research, CRISPR/Cas is widely seen as one of the most promising approaches to making genetic alterations that might benefit human health—I focus on human health research here. Potential applications include genome editing for (i) the treatment of monogenetic diseases like cystic fibrosis based on a mutation of the CFTR gene (Veit et al., 2016), (ii) the treatment of polygenetic and multifactorial diseases like Alzheimer's dementia *via* intervention on APP, PSEN1, and PSEN2 (Bekris et al., 2010), and (iii) reducing the risk of polygenetic and multifactorial diseases, e.g., reducing dispositions for breast and ovarian cancer *via* intervention on BRCA1 and BRCA2 (Kuchenbaecker et al., 2017).

Several technical obstacles come along with genome editing in general and heritable editing in particular. It is important to mention these technical problems here, because the majority of experts on the CRISPR/Cas technology currently lean toward a clinical moratorium on heritable human genome editing, which would leave room for basic research on these technical issues. Also, finding technical solution to solve or cope with these problems is considered a necessary requirement for a responsible transition to clinical research on heritable genome editing. I highlight just a few of the issues currently discussed:

1. Off-target editing: CRISPR/Cas9 sometimes edits genetic sequences other than the intended sequence (identified by the guide RNA sequence). The error rate of specific applications of endonuclease complexes is an object of current research (Cho et al., 2014; Lin S. et al., 2014; Zhang et al., 2015; Park and Beal, 2019).
2. Genetic mosaicism: Genome editing in a zygote or an early embryo comes with a significant chance that some of the cells in the resulting organism will not have the desired edit (Mehravar et al., 2019). Having two or more genetically different sets of cells in one's body might result in health issues (Biesecker and Spinner, 2013).
3. On-target effects: Some genes that cause serious genetic diseases also give carriers some protection against infectious diseases when the gene in question is present in one copy. A paradigmatic example is the HBB gene. If someone inherits two copies of HbS (mutated version of HBB) from both parents, then this individual will suffer from sickle cell anemia. Yet if someone only inherits one copy of HbS from either parent (or the mutation occurs naturally), then this patient will suffer from less dramatic health effects and gains some protection against malaria (Archer et al., 2018).
4. Ability to select appropriate gene targets: Due to our currently limited knowledge of human genes, genetic variation, and interactions between genes and the environment, it is not clear whether we are in a position to make a well-justified decision on appropriate gene targets (National Academies of Sciences, 2017) and avoid or minimize risks like on-target effects.
5. Access to and pricing of clinical medical treatments: One major worry is that medical treatments based on CRISPR could be extremely expensive and thus not broadly available for patients in the long term. There are several reasons for extreme pricing of novel gene therapies, including the necessity to recoup the development costs, higher effectiveness of novel therapies compared to other treatments as well as technical challenges of production and delivery in clinical practice (Wilson and Carroll, 2019).

These and other technical and ethical issues have led to a general hesitancy with regard to heritable genome editing in clinical practice which would involve genome alterations in human embryos and the birth of genetically altered humans (Brokowski, 2018).^4^

## An Epic Scientific Misadventure

One factor that majorly contributed to the current hesitancy with regard to heritable human genome editing and in particular clinical applications of the CRISPR/Cas technological is the research scandal that has unfolded around the Chinese biophysicist He Jiankui between 2018 and 2020. The case has been widely commented (Greely, 2019), although crucial details on experimental procedures, financial connections to companies within the biotechnology and biomedical sector as well as support from political and scientific intuitions remain unclear—not to mention individual support from scientists who were in contact with He (Cohen, 2019b).

In the following, I will briefly explain the scientific background of He's experiment, which Francis Collins dubbed an “epic scientific misadventure,” give a rather coarse-grained overview on the timeline of events, and then provide an overview on the legal and moral fallout (Cohen, 2018b).^5^ More fine-grained reconstructions of the events between 2018 and 2020 can be found in various recently published sources (Baylis, 2019a; Greely, 2019, 2021; Kirksey, 2020). The description of this case illustrates the negative effects resulting from the presence of secondary interest, which gave rise to recent efforts to regulate human germline research with CRISPRS/Cas and similar technologies. Since some of those interests could also present in elite scientists who make a regulatory effort regarding heritable genome editing, one could make an argument from analogy for a more cautious stance toward the role of leading scientists in policy making.

He obtained his PhD in 2010 under Michael W. Deem at Rice University and subsequently worked on CRISPR/Cas9 gene-editing as a postdoc under Stephen Quake at Stanford University. After returning to his country of origin (People's Republic of China) in 2012 within the Thousand Talents Program (TTP),^6^ he was employed at the Southern University of Science and Technology (SUSTech) in Shenzhen, Guangdong province, China. There He was in charge of a lab funded by the Chinese government and received 1 million yuan (around 144.000 USD) in angel funding (high risk funding for start-ups) for new companies from TTP as well as other funding from private investors (Kirksey, 2020). Such financial ties as well as working conditions, which encourage novel and commercially interesting research projects, are commonly found top tier research institutions. Thus, it is unsurprising that He founded at least two companies. Direct Genomics,^7^ based in Shenzen (founded in 2012), is concerned with the development of a single-molecule sequencing device based on a technology previously developed by Stephen Quake and formerly licensed by Helicos Biosciences.^8^ Vienomics Biotech was founded in 2016 and offers genome sequencing and screening for cancer patients and at-risk groups.

Relatively unknown within the Western scientific community, He announced on November 25, 2018 that he had successfully edited the genome of two embryos using *in vitro* fertilization and CRISPR/Cas9. He had targeted CCR5, a gene that is essential for HIV-1 to induce its viral DNA into cells. His experiment was based on the observation that a deletion of 32 base pairs in CCR5 on chromosome 3 is responsible for a resistance to HIV-1 infection (Samson et al., 1996). Such a deletion, called CCR5-Δ32, results in the production of non-functional copies of the CCR5 protein found on the surfaces of T-cells, which are white blood cells in the immune system. Humans with two dysfunctional copies of CCR5 are virtually resistant against HIV-1 infections, since HIV-1 viruses cannot establish a connection to T-cells with a crippled form of CCR5 (Brelot and Chakrabarti, 2018). However, even individuals with two dysfunction copies of CCR5 can still contract an infection with HIV-2.

He's announcement came as a huge surprise and shock to the scientific and medical community, because it was also announced that resulting from this experiment two genetically edited babies had been born—pseudonymized as Lulu and Nana (Normile, 2018). The parents participating in this particular experiment were couples where the male was HIV positive and the female was HIV negative. One day later, on November 26, 2018, SUSTech distanced itself from the experiment and declared that He was on leave since February 2018 and that the experiment was not affiliated with SUSTech. The experiment contradicted SUSTech's codes of conduct for biomedical research and He therefore lost his position as an associate professor at SUSTech (Normile, 2019a). On November 28, 2018; He gave a talk on his experiment at the Second International Summit on Human Genome Editing in Hong Kong, which was met with almost exclusive rejection by the audience—more on that later.^9^ On December 1, 2018; He as well as his family were put under house arrest and detained in a guest house of SUSTech in Shenzen, guarded by government agents. After 1 year, on December 30, 2019—during the ongoing international debate on the legal and ethical implications of this case—He as well as two other scientific collaborators were convicted by the Shenzhen Nanshan District People's Court. He was sentenced to 3 years in prison for illegal medical practice and also fined 3 million yuan (US $465,000/390,000 EUR). His colleagues involved in the experiment received lesser prison sentences and fines. Zhang Renli received a 2-year prison sentence and was fined 1 million yuan (US $144,000/130,000 EUR. Qin Jinzhou was sentenced to 18 months in prison and fined 500,000 yuan (USD$ 72,000/65,000 EUR) (Normile, 2019b; Cohen and Normille, 2020; Cyranoski, 2020; Kirksey, 2020). On January 1st, 2020, Chinese news agency Xinhua announced this verdict and also reported that a third baby was born.^10^ Currently, there is no information on the health condition of the children available.

The experiment was almost unanimously condemned as immoral, scientifically premature, probably illegal in the People's Republic of China, and a general failure of scientific self-regulation. In an early case description, legal scholar and bioethicist Henry Greely thus called it a “reckless ethical disaster” and “fiasco” (Greely, 2019). Types of criticism regarding this case are three-fold. They include objections pertaining to (i) a lack of transparency regarding scientific and organizational aspects of the case, (ii) bioethical issues (e.g., a lack of medical necessity due to the availability of alternative methods for conceiving healthy offspring, misclassification of the experiment as a treatment, etc.) as well as (iii) the experiment's unlawfulness and the general disregard for protocol in biomedical research expressed by it. The following list of items comprises just some of the issues with the experiment that are currently discussed in medical ethics and research ethics, it is meant to give the reader an idea of the magnitude of the violations against good medical and scientific practice:

1. *Inappropriate consent form*: The 23 page long informed consent form is written in very technical language and includes no discussion of off-target effects or undesirable on-target changes. By not mentioning a common method applied in the context of intrauterine insemination and *in vitro* fertilization in cases in which one partner is HIV positive, He intentionally or recklessly depicted the experimental and more risky treatment as the favorable alternative. Furthermore, the form failed to provide information about alternative methods of preventing an HIV infection. The consent form was not approved by an institutional review board, either. Finally, staff members without specific training took only 120 min to explain the experiment to participants (Greely, 2019; Jonlin, 2020; Kirksey, 2020; Shaw, 2020).
2. *Lack of transparency*: He bypassed peer review by announcing the result of the experiment in a video posted on youtube.com on November 25, 2018.^11^ He provided no research paper explaining the exact experimental procedure and results of the experiment. It is still unclear (September 2021), but based on screenshots from his presentation at the Human Genome Editing in Hong Kong, one can assume that only one of the two siblings has two copies of CCR5 edited, while the other sibling still has a functional CCR5 gene. Therefore, one of the siblings can still be infected with HIV (Cohen, 2018a). The health status of the third child, which resulted from an experiment with a different couple, is currently unknown.
3. *Violations of research protocol*: The experiment was neither registered before the clinical research was done, nor thoroughly checked or approved by an independent ethical review board. He forged ethical review papers in order to enlist volunteers for the procedure (Normile, 2019b), and had raised his own funds, deliberately evading institutional oversight.
4. *No medical necessity*: The immunization *via* CRISPR/Cas-based germline intervention against HIV infection was not a medical necessity, since alternative medical procedures to prohibit an infection with HIV exist and are routinely employed in *in vitro* fertilization, e.g., sperm washing (Savasi et al., 2007; Zafer et al., 2016; Carvalho et al., 2021).
5. *Illegal medical procedures*: He used sperm washing in order to separate sperm from sperm fluid, which contained HIV viruses. Yet IVF procedures in general and sperm washing in particular are currently banned in China for HIV infected couples. This is also problematic, since offering the participation in such an experiment can be seen as a strong incentive for HIV infected couples or gay couples, wishing a healthy and genetically related offspring without having heterosexual intercourse for the sake of procreation.
6. *Problem of target selection*: He target CCR5 for genome editing, although CCR5 has a protective role in immune reactions against the West Nile virus, which is common in Europe, Africa and North America (Kohlmeier et al., 2008; Cyranoski, 2018a), and a CCR5 deficiency predisposes to fatal outcome in influenza virus infections (Falcon et al., 2015).^12^
7. *Not a medical treatment, but genetic enhancement*: CCR5-Δ32 might, in addition to establishing a resistance against infections with HIV-1, even enhance certain cognitive dispositions, since CCR5 is linked to improved memory function in mice (Zhou et al., 2016) as well as enhanced recovery from strokes and traumatic brain injuries in humans (Joy et al., 2019). More fundamentally, not having a rare favorable genetic disposition is obviously not a disease, thus creating this genetic disposition is not a medical therapy in a strict sense of reducing or eliminating disease, but rather an instance of enhancement resulting in risk reduction.
8. *Failure to provide appropriate health care provisions*: The children whose genetic material has been altered will learn at some point in their life that they are the result of an experiment in heritable human genome editing, yet the provision of psychological and pedagogical support for the family was not taken into consideration. Also, if the observation under point 1 is correct, one of the siblings can still be infected with HIV.

It can be assumed that He anticipated at least some of this criticism, since he published a research paper in *The CRISPR Journal* in 2018 (Jiankui et al., 2018), which was retracted due to the circumstances surrounding this case and a lack of full and open disclosure of conflicts of interest on November 26, 2018. This paper encouraged “[…] the scientific community to support the public in making informed decisions about gene surgery's clinical utility, limitations, risks, regulatory needs, and future role in society” (Jiankui et al., 2018, p. 2). The authors in particular formulate five core principles for gene surgery in human embryos, including mercy for families affected by heritable diseases, restriction of gene surgery to the prevention of serious diseases, respect for child's autonomy, rejection of genetic determinism, and equal access to gene surgery (Jiankui et al., 2018, p. 2). It is challenging to not conceive this contribution as a *post-hoc* attempt to rationalize the experiment and create a flimsy impression of moral integrity and social responsibility, especially given the lack of disclosure of the experiment in this publication. Also, it is quite astonishing that this contribution sustained the peer review process, since it barely refers to the bioethical debates regarding heritable human genome editing (see Getz and Dellaire, 2020). Against the main thesis of this investigation, Jiankui et al. (2018) can be seen as a rather obvious example of an attempt to influence the public debate on the moral acceptability of clinical research on gene surgery, which brings about a heritable change of a human germline.

Commentators highlight three main motivational factors for He's experiment, listed here in random order: (i) He worked in an environment that provided strong financial incentives, as he received angel funding from TTP as well as a yet not fully identified amount of private funding for his laboratory, private companies, and future business endeavors (Coleman, 2018; Baylis, 2019a; Qiu, 2019; Kirksey, 2020; Greely, 2021). (ii) He had strong career ambitions and—according to many of those who corresponded with him before his detention—wanted to be the first in creating genetically altered human beings (Belluck, 2017; Greely, 2021). (iii) Furthermore, due to his experience with the suffering of HIV and AIDS patients in China, he seemed to have had genuine sympathy with patients who might benefit from his research.^13^

The case is now inextricably linked to the development of CRISPR/Cas (Baylis, 2019a; Kirksey, 2020; Davies, 2021; Greely, 2021; Isaacson, 2021) and a paradigmatic example of a rogue scientist who, due to immense interests in scientific reputation and vested commercial interests, circumvented laws and bioethical standards. In the context of this contribution, this case serves to make the urgency of establishing effective regulation obvious. It will also make it at least initially plausible that further regulations to cope with commercial conflicts of interest as well as conflicts of commitment are needed, as the identified motivational factors (i–iii) suggest.

## Experts in Moral Debates on the Ethical Issues of Crispr/Cas Technology and Policy Making

Moral worries on the matter of human germline editing and calls for a broad societal discussion on the bioethical issues predate the He Jiankui case. In fact, the debate about the ethics of human genome editing can be traced back to the debate on eugenics movements in the 1950s (Kevles, 1985). Yet, it took until the 1970s for scientists to imagine genetic interventions on an individual level, which go beyond the restriction and encouragement of certain patterns of procreational behavior. This development was stimulated by new research on restriction enzymes and recombinant DNA and led to the 1975 Asilomar Ban on recombinant DNA technology (Berg et al., 1975). With the rise of bioethics in the 1980s, bioethicists took then newly established ethical frameworks, in particular the principlism developed by Beauchamp and Childress (2001), and considered germline editing by appealing to the principle of beneficence and non-maleficence (e.g., Fletcher and Anderson, 1992):

“*[…] searches for cure and prevention of genetic disorders by germ-line therapy arise from principles of beneficence and nonmaleficence, which create imperatives to relieve and prevent basic causes of human suffering.” (Fletcher and Anderson*, *1992**)*

Generally speaking, the debate on human germline editing after the development of technologies for genetic engineering, which allow for target specific genome interventions, was for the longest time focused on the transition from basic to clinical research and considered attempts to change the genome of human embryos as a hypothetical scenario. Yet, after a team of scientists from China (Liang et al., 2015) announced that they had used CRISPR/Cas9 to edit human tripronuclear zygotes, new efforts were taken to prohibit premature heritable genome editing. Further instances of the debate on gene surgery and heritable human genome editing include, in particular, subsequent statements made by various science organizations (The National Academies of Sciences, Engineering and Medicine, 2017). Inter alia, the German National Academy of Sciences Leopoldina in cooperation with other scientific organizations in Germany wrote in 2015:

*It is important to have an objective debate that informs all stakeholders in a clear and transparent manner about the status of research and development into the techniques, and to ensure that any decisions taken are based on sound scientific evidence. (National Academy of Sciences Leopoldina et al.*, *2015**)*

The scandal surrounding He Jiankui has thus fueled, but not initiated two debates which were already present in bioethics, but used to be a hypothetical scenario. Since He's “epic scientific misadventure,” the scenario is now conceived as an imminent reality and thus, a top priority. Therefore, the *debate on a moratorium on heritable human genome editing* gained traction right after He's talk at the Second International Summit on Human Genome Editing (Cohen, 2019a; Davies, 2019; Dyer, 2019; Hough and Ajetunmobi, 2019; Konig, 2019; Lander et al., 2019; Macintosh, 2019; Wolinetz and Collins, 2019). Currently, many scientific, juridical and administrative issues are under discussion. Regarding the scope of a moratorium, leading scientists seem to lean toward a moratorium with regard to clinical studies on human germline editing, which leaves open the possibility to do basic research on technical aspects of CRISPR/Cas in basic research (Lander et al., 2019; Wolinetz and Collins, 2019). The latter is seen as necessary to engage in well-informed risk-benefit analyses fundamental to a translational pathway toward clinical applications. Another issue is the precise way to implement a global moratorium, e.g., *via* an exclusion from funding sources, outlawing certain types of research or self-imposed restrictions. Also, due to the relatively ready accessibility of the CRISPR/Cas technology, it is unclear how compliance with a moratorium might be enforced in private companies and countries without national regulatory frameworks on human genome editing or where an institutional structure is missing. From a philosophical point of view, there is the question of how a moratorium is compatible with commonly shared values of scientific freedom (Wilholt, 2010, 2012) and what the relevance of any actual hindrance of scientific progress might be (Konig, 2019; Macintosh, 2019). While the demand for a moratorium is certainly understandable, the justification for a moratorium on heritable human genome editing (or other scopes of a moratorium) would have to show that the case for a moratorium is stronger than the combined justificatory power of well-established arguments for positive and negative types of freedoms assembled under the generic concept of freedom of science. The latter pertain to, e.g., research freedom as a derivative of intellectual autonomy, its political value and epistemic utility (Wilholt, 2010). An ill-justified moratorium could potentially infringe on fundamental liberty or political rights.

The other debate that has been impelled in the wake of He's experiment concerns the exact criteria of a *pathway toward different types of clinical applications*. This debate relates heritable human genome editing to a whole range of bioethical issues, including the usage of human embryonic stem cells and products of synthetic biology like cell-based models of embryos or embryoids (Aach et al., 2017). Many national ethics councils and committees currently seem to agree with the following requirements (Brokowski, 2018; Baylis et al., 2020): (1) No human germline editing should be tried until risks and benefits are sufficiently known. (2) More time for ethical debates and establishing national and international legal framework on the editing of chromosomal and mitochondrial genetic information is required (Lander et al., 2019). (3) A broad societal discourse informed by scientists, moral and theological scholars is necessary. Finally, (4) societal consent could be necessary to adopt a positive stance toward certain types of clinical applications. It seems possible that some types of genome therapy which would affect the human germline, such as the treatment of some severe heritable monogenetic diseases, might find wide public acclaim in many societies (given that the risk-benefit ratio is positive).^14^

It is within the debate on a translational pathway to human genome editing that scientific experts on the CRISPR/Cas technology exercise particular influence. They take on crucial roles in establishing an international framework and helping to develop national policies (Baylis, 2019a). Typical functions experts take on in this context include (a) expert consulting in policy making processes, for instance by appearing in public hearings or writing scientific reports on risks and benefits of specific applications of the CRISPR/Cas technology. (b) Experts also serve as moderators and adopt a guiding function in initiating and maintaining a dialogue on ethical issues of the CRISPR/Cas technology. This currently often happens in semi-public formats, for instance after workshops and conferences, when renowned experts write scientific statements concerning the grant policy strategies they deem fit to find a purported balance between scientific freedom and respecting other ethical values. More recently, philosophers have begun to criticize such forums, because they are in stark contrast to the idea of a clear and transparent debate which includes all stakeholders—and not just scientists working with CRISPR/Cas (Stengers, 2018; Baylis, 2019a). (c) Experts engage in science communication by providing laypersons with the empirical knowledge about the CRISPR/Cas technology necessary to address the ethical issues. (d) Finally, experts engage in public advocacy for specific policies. This function is often considered unproblematic both in the debate on a moratorium on CRISPR/Cas and the debate on a translational pathway. The worry is that leading experts in the field of CRISPR/Cas could be affected by conflicts of (commercial) interest and conflicts of commitment. More concretely, if scientists have founded biomedical companies, have strong interest in peer recognition as well as a character defining urge to understand the nature and possible applications of CRISPR/Cas, then efforts undertaken by them to explain CRISPR/Cas-based genome editing, prospective applications as well as risks and benefits in clinical practice could intentionally or unintentionally foster their own research interests and moreover accommodate their recognitional or financial interests. Finding evidence for this concern is in my view extremely demanding and in the following I make a case for a more cautious stance toward the role of experts in this debate, due to our inability or limited ability to rule out conflicts of interest and conflicts of commitment. The train of thought here is that ignorance in conflicts of interest and conflicts of commitment of experts implies the adoption of less trust in the impartiality of those experts. I explicitly do not insinuate any form of corruption among these leading experts.

The previously presented overview on functions of scientific experts on the CRISPR/Cas technology in public and semi-public debates as well as policy making shows the ways in which leading scientists working as ethics architects and issue advocates (Baylis, 2019a) can gain a high degree of *intrinsic influence in public and semi-public debates and regulatory processes*, insofar as they serve as consults and moral authorities, but also *extrinsic influence* on the organizational features of public and semi-public debates. An example for intrinsic influence in debates can be seen in the linguistic framing of the debate on a translational pathway. Jennifer Doudna speaks about a “responsible pathway,” “a viable path toward responsible use” and “a prudent way forward” (Baltimore et al., 2015; Doudna, 2019). As a Nobel prize winner, she has more opportunities to frame the problem in these terms and receives more attention, compared to critics. Also, when the debate is framed as the search for a responsible use, the basic question of whether there is a responsible use at all is almost off the table. A typical example for extrinsic influence are conferences (e.g., the International Summit on Human Genome Editing), which are organized as semi-public events and are generally not suitable for a broad societal discourse with many stakeholders. Also, the currently held public forums for discussing the ethical implications of CRISPR/Cas are often organized by scientists who have control over the selection and influence of participants, be they religious leaders, patients' and disability rights activists, social scientists, legal scholars or governmental representatives (Doudna and Sternberg, 2017). For instance, the agenda for the Third International Summit on Human Genome Editing (to be held in March 2023 at the Francis Crick Institute in London) reveals a number of speakers working on bioethical issues.^15^ Yet, it is unclear whether those experts representing special interest groups will actually participate in the formulation of a final statement regarding ethical aspects of clinical applications. Also a ratification by the participants of a final statement on ethical issues is currently not planned, thus one needs to assume that any ethical assessment results from the internal deliberation of the organizers.

This high degree of intrinsic and extrinsic influence of a handful of individuals might be concerning in and of itself. When it is paired with commercial conflicts of interest as well as conflicts of commitment, it certainly poses a serious threat to the epistemic and moral integrity of decision-making processes in this context. Research has shown commercial conflicts of interest in biomedical research to be epistemically corrupting factors in research and publication processes^16^ as well as in policy making and the development of clinical and research guidelines (Hakoum et al., 2020; Nejstgaard et al., 2020; Tabatabavakili et al., 2021).

## A Plea for Caution

In the context of the regulation of the CRISPR/Cas technology, not much attention is currently directed at commercial conflicts of interest and conflicts of commitment among biomedical researchers. The scientific community is presently rather occupied with the real possibility that other rogue scientists emerge. The concern about individuals surging forward on human germline editing has been further stoked by an announcement of molecular biologist Denis Rebrikov in 2019 (Cyranoski, 2019a,b), who is currently exploring the possibility to edit a gene linked to deafness (GJB2) with the help of CRISPR/Cas. Rebrikov is employed at Pirogov Medical University in Moscow and one can assume that such an experiment would be illegal in Russia, since the Russian federal law on biomedical cell products from 2016 bans the production of human embryos for research purposes and their implantation (Matthews and Moral, 2020).^17^ As unsettling as such an announcement may be, it is dangerous to let (upcoming) scandals concerning individual deviant researchers detract from the risks that spring from the influence scientists exercise on public debate within the bounds of current regulations.

Above, I indicated that a mixture of career aspirations, commercial interests and sympathy with HIV/AIDS patients was likely the motivational background for He and his colleagues' violation of Chinese law, bioethical guidelines, and principles of good scientific practice in their experiment on CRISPR/Cas-based human germline editing. Inasmuch as these factors are actually good explanations for the blatant misconduct that has occurred in this case, any motivational setup in scientific experts who exhibit a comparable pattern of career aspirations, commercial interests and strong personal ideas about medical priorities must be considered a risk factor for compromised judgment in context of public and semi-public debates as well as policy making. This leads us to two *unsettling questions*: (1) Do we have reason to believe that outspoken public advocates for a specific type regulation on genome editing technologies have conflicts of interest and conflicts of commitments? (2) What are the risks resulting from conflicts of interest and conflicts of commitment in CRISPR/Cas policy making?

There are at least two reasons why we trust leading scientists and give them intrinsic and extrinsic influence on our discursive culture and various policy making processes. For one, we generally have trust in the various systems brought into place to designate academic rank, give scientific credit and acknowledgment for scientific achievements. These systems include, e.g., academic qualification systems (undergraduate programs, graduate programs etc.), peer review systems in journals, science award committees, and organization committees of scientific workshops and conferences. Generally, we trust these systems—or the individuals behind these systems—and assume that they correctly assign academic credentials and ranks within the organizational structure of scientific institutions. Secondly, there is also a tendency to assume that a high degree of scientific acknowledgment by scientific peers for an individual scientist also signals a certain integrity in that person, or even moral expertise with regard to her research field. In the following, I want to challenge our somewhat unconditional trust in experts by pointing toward crucial issues with commercial conflicts of interest among CRISPR/Cas experts.

Information about conflicts of interests and conflicts of commitment among experts on CRISPR/Cas engaging in the debate about its regulation is not easily accessible. It is often difficult to find information about the precise nature of conflicts of interest, including financial compensation. In the following, I will focus on the example of Jennifer Doudna, because she is one of the inventors of the CRISPR/Cas technology and thus one of the leading experts in this field. She is also actively involved in public debates on the ethics of CRISPR/Cas and has been for at least 8 years, highlighting the importance of a broad societal debate and a “thoughtful approach” to human genome editing. She is pleading for a moratorium on clinical applications of CRISPR/Cas and argues for strong national regulations as well as harsh sanctions against those who violate established policies—e.g., at minimum a loss of funding and publication privileges (Doudna, 2019). Doudna also has multiple financial ties to pharmaceutical companies, she has founded companies working with CRISPR/Cas and serves on corporate scientific advisory boards. On her laboratory's website, she lists several conflicts of interest. The subpage can be found on the bottom section/footer of the page, an area commonly reserved for copyright information, sitemaps, privacy policies, terms of use and contact details (see footer on https://doudnalab.org/, retrieved 08-25-21), which can be readily ignored by users. Information on conflicts of interest is not presented in detail in her curriculum vitae. In her short bio, there is only this rather non-descript hint:

“In addition to her scientific achievements, Doudna is also a leader in public discussion of the ethical implications of genome editing for human biology and societies, and advocates for thoughtful approaches to the development of policies around the safe use of CRISPR technology. Doudna is an investigator with the Howard Hughes Medical Institute, senior investigator at Gladstone Institutes, and the President of the Innovative Genomics Institute. She co-founded and serves on the advisory panel of several companies that use CRISPR technology in unique ways.” (https://doudnalab.org/bio/, retrieved 08-25-21).

The information on the website identifies her as a cofounder of Caribou Biosciences, Editas Medicine, Scribe Therapeutics, Intellia Therapeutics and Mammoth Biosciences. In addition to this, she is also a scientific advisory board member of Vertex, Caribou Biosciences, Intellia Therapeutics, eFFECTOR Therapeutics, Scribe Therapeutics, Mammoth Biosciences, Synthego, Algen Biotechnologies, Felix Biosciences, The Column Group and Inari. Furthermore, she is a Director at Johnson and Johnson and Tempus, and her research projects have been sponsored by Biogen, Pfizer, AppleTree Partners, and Roche. It is important to highlight here that unlike other elite scientists who made fundamental contributions to CRISPR/Cas, Doudna actually declares commercial conflicts of interests in a semi-transparent way on her website. Emmanuelle Charpentier's website, for instance, only includes links to CRISPR Therapeutics and ERS Genomics—two companies she co-founded.^18^ Fang Zhang's Website only mentions that he is a founder of Sherlock Biosciences and the public companies Arbor Biotechnologies, Editas Medicine, and BEAM Therapeutics, yet tangible details about financial interests are not available.^19^

This reveals a situation in which secondary interests are present, but in which there is no direct, centralized way to quantify the magnitude of these interests. For sure, secondary interest are not by definition illegitimate, but rather a natural part of professional agents' life in complex socio-cultural and economic settings. Prospects of commercial applications can also be a part of a well-reasoned justification for a specific research agenda and policy decision on CRISPR/Cas technology. The issue lies elsewhere. If conflicts of interest and commitment of experts who engage in public debates are not declared or declared in an uninformative way, then participants in these debates have incomplete knowledge on the motivational background for experts' stances on the issues that are being debated. Thus, participants are not well-informed when agreeing or disagreeing with approaches relating to matters like a moratorium or regulatory efforts toward clinical applications. In particular, they lack background knowledge about reasons to inquire into the nature of some expert's contribution to the debate: they may overlook an occasion to wonder whether they are listening to a relatively disinterested expert *explaining* the CRISPR/Cas technology or to a speaker who is heavily invested in commercial endeavors relying on this technology and intends to make a case in a scientific priority dispute.

In her book—written together with Michael H. Sternberg—on the development of the CRISPR/Cas technology, Doudna is quite clear about her reservations concerning editing the human germline (Doudna and Sternberg, 2017). In a chapter on curative applications of CRISPR/Cas she writes:

“*I am extremely excited and enthusiastic about virtually all the phenomenal progress being made with CRISPR—save for the advancements on one front. I think we should refrain from using CRISPR technology to permanently alter the genomes of future generations of human beings, at least until we've given much more thought to the issues that editing germ cells will raise. Until we have a better understanding of all the attendant safety and ethical issues, and until we have given a broader range of stakeholders the opportunity to join the discussion, scientists would do well to leave the germline alone. But, really, whether we'll ever have the intellectual and moral capacity to guide our own genetic destiny is an open question—one that has been on my mind since I began to realize what CRISPR was capable of. For this reason and others, I've come to see a clear boundary between the procedures described in this chapter and those involved in germline editing. We should think twice before crossing that line. And then we should think again.” (Doudna and Sternberg*, *2017**)*

A careful reader of Doudna and Sternberg (2017) will certainly have the impression that Doudna is honestly interested in the responsible advancement of the CRISPR/Cas technology for the sake of humanity. Other sources suggest that she was even morally appalled by He's experiment (Cyranoski, 2018b). Yet, her public talks about the CRISPR/Cas are more focused on the development and functioning of the CRISPR/Cas technology as well as medical and commercial prospects. Ethical issues are usually mentioned as such, but not elaborated in detail.^20^ This is problematic, because in shorter statements Doudna directs the public debate about ethical implications of the CRISPR/Cas technology to certain outcomes without engaging in the details of the bioethical debates (Doudna, 2019) which concern, for instance, the usage of human embryos, embryonic stem cells and animal experimentation. Yet, she is considered by the public as one of the experts on the *ethics* of CRISPR/Cas and thus has access to public forums.^21^

This is reason enough to think that at least some of the leading experts in the CRISPR/Cas technology are in a situation which combines (i) a high level of expertise in scientific and clinical aspects of the CRISPR/Cas technology, which is relevant for the moral discourse, paired with (ii) self-declared commercial conflicts of interest (Greely, 2021) and (iii) a strong influence on public understanding of CRISPR/Cas as well as debates on the regulation of this technology. For instance, leading experts have the opportunity to publish opinion pieces in top-tier scientific journals and other media outlets, give plenary talks and television interviews. In the following I will explain why such a situation can introduce severe bias into the discourse on the ethical implications of CRISPR/Cas.

There are several ways in which experts in the CRISPR/Cas technology can influence public discourses and policy making processes and thereby might bring their research and commercial interests to bear on any international framework to be developed for genome editing. (a) Experts can advocate for a moratorium with regard to clinical studies of CRISPR/Cas-based human germline editing and highlight the importance of basic research on the safety and efficiency of CRISPR/Cas. This can be done without reacting to critics like (Guttinger, 2018) who point out that the ultimate proof of safety and efficiency of CRISPR/Cas-based human germline editing must be done in human *in vivo* and cannot be figured out in basic research. (b) The stipulation that a responsible pathway toward clinical applications is the only option that reconciles scientific progress and ethical concerns (Baylis, 2019b; Hurlbut, 2019) avoids the question of principle with regard to human germline editing. (c) Focusing on prospects of human genome editing, like cures for diseases and clinical applications within the next 10 years disregards the fact that developments in other fields in biomedical research suggest that translation time is probably much longer. For instance, after several decades of research, we only have one FDA reviewed and approved clinical therapy based on human stem cells, hematopoietic stem-cell transplantation (HSCT) (Felfly and Haddad, 2014; Mahla, 2016). (d) A persistent positive linguistic framing of the issue, in particular the normative enhancement of a neutral concept like “translational pathway” by speaking about a “*prudent* way forward” or “*responsible* pathway” is conducive to the conception that a safe translational pathway is possible and preferable to a permanent moratorium. Also, the former seems to require just the bare minimum of risk-assessment based on basic research about the CRISPR/Cas technology. (e) A voluntarily or involuntarily induced moral fallout, which leads from the alleged necessity to gain knowledge about specific aspects of the CRISPR/Cas technology (see point b) to the moral acceptability of the usage of human embryos and human stem cells in basic research on CRISPR/Cas without engaging in the deep and complicated ethical issues with this practice (Devolder, 2015). The same is valid for the moral acceptability of synthetic human-like entities with embryo-like features in basic research. (f) Another problematic issue is that scientists can simply select and promote an ethical framework which creates a window of opportunity for their research, without seriously engaging in the ethical reasoning behind it. This is a problem which commonly arises when scientific methods are morally problematic and their application requires an ethically well-reasoned justification. For instance, in basic research on off-target editing and other methodological aspects of the CRISPR/Cas technology, animal experiments are currently considered a step toward research on human genome editing. Animal experiments in general are widely criticized for their lack of objectivity and lack of moral justification. Now, it is certainly possible to pseudo-justify animal experimentation in basic research on CRISPR/Cas without seriously considering the moral wrongness of animal experiments or arguments against animal experimentation. For example, in a recent book on so-called animal research ethics, which was prominently featured in 2020 in Science (Grimm, 2020), Beauchamp and Grazia assume from the beginning that advocates of strong animal rights—those who reject the idea that the suffering of nonhuman animals in involuntary experiments is the sort of thing that can be outweighed by expected social benefits—are not “reasonable” and “open minded” (Beauchamp and DeGrazia, 2020). It is all too easy for scientists to simply adopt such an ethical framework as a pro forma stance, since it suits research interests, without considering the arguments against such a framework.

These hypothetical examples suggest that commercial conflicts of interest and conflicts of commitment, such as the economic success of your industry partners or a strong epistemic desire to find an answer to a research question, constitute a risk in public and semi-public debates as well as in policy making. These interests could bring scientists to make a case for a policy or a more general research framework which primarily suits their interests. Although not an example for outright corruption, these practices can still can be considered *manipulative* and warrant a more cautious stance toward the influence of leading experts.

In addition to these ploys, I will bring forward three further arguments to raise concerns with regard the influence of experts in public advocacy and policy making: First, since information on commercial conflicts of interest of leading experts is sometimes not readily available or declared in an uninformative way (leaving out precise financial information, etc.), our ability to assess the validity of advocated stances on a moratorium and a translational pathway is equally limited. This situation is inacceptable, especially since the CRISPR/Cas technology is a step toward changing the shared heritage of humanity. If the talk of a broad and transparent societal discourse on human genome editing has any meaning, then it must include informational transparency with regard to the commercial interests of scientists who exercise their right and their responsibility to participate in this discourse.

Second, the reliance on leading experts on the science of CRISPR/Cas in public debates and policy making to clarify ethical issues is also in conflict with philosophical insights into important differences between scientific expertise and moral expertise as well as deference to experts of either type. While experts on CRISPR/Cas are absolutely essential in helping laypeople understand the foundations and applications of this technology, it is far from obvious why we should regard them as experts in the ethical issues associated with CRISPR/Cas and defer to their moral decisions about these issues. For instance, empirical and methodological knowledge on CRISPR/Cas is certainly highly important in correctly reconstructing, evaluating and deciding a moral problem like the case for a moratorium. Yet, empirical and methodological knowledge—say, about off-target events or on-target effects—does not imply any superior capacity to justify a certain weighing of the associated risks and potential benefits or a capacity to frame the issue as a case of risk-benefit analysis in the first place.

A final issue is that experts on CRISPR/Cas may achieve relatively high influence on public debate and decision making due to their standing within the academic system, their relationships to private companies and political decision makers—yet they lack a public mandate. First-rank experts meet virtually no resistance in gaining access to public and semi-public debates. However, given the reasons presented in this section, it seems that we should meet them with not an especially high, but perhaps even reduced initial trust when it comes to their ethical assessment of the procedures in question. In any case, we should require more initial information on possible corrupting factors, even when we at the same time trust their epistemic and methodological assertions owing to their academic credentials.

## Toward More Transparency

What precautionary measures should we adopt in the face of these problems? There are at least three types of measures that could promote the integrity and political legitimacy of decision-making processes and public debates on the regulation of the CRISPR/Cas technology.

First, we need scientists to disclose information on conflicts of interest publicly and in more detail. One recent example of an attempt at such a central registry is a platform which already enables journalists and interested citizens to acquire information about commercial conflicts of interest. The *Dollars for Profs Project* by Sisi Wei, Annie Waldman and David Armstrong from ProPublica was started on December 6, 2019 (https://projects.propublica.org/dollars-for-profs/, retrieved 01-09-2021). This system is a great tool in figuring out commercial conflicts of interest, yet it is vastly incomplete. It lists information obtained from the National Institutes of Health *via* public record request filed at multiple public state universities. Yet, many universities decline to reveal conflicts of interests of their scientists.^22^ ProPublica is a newsroom which intends to help investigative journalism in the public interest in the US. Thus, it lacks both the scientific legitimacy of other types of registries, for instance, state funded registries on clinical trials, as well as the necessary worldwide coverage. Information on conflicts of interest obviously has to be made available in a more comprehensive and scientifically established way. One way in which this could be done might be by having the WHO found a publicly available registry on conflicts of interest for researchers. In addition to this, research funding agencies could make it mandatory to register conflicts of interest and conflicts of commitments in this registry, the data being updated on a yearly basis.

Second, we need to change our stance on high-profile experts and their access to public debates. The declaration of conflicts of interest and conflicts of commitment should also be a requirement for access to large audiences, which need this information prior to talks in order to understand the proper economic context of certain policy positions. For instance, a TED Talk from a leading expert in CRISPR/Cas should include a disclaimer of the speaker's commercial conflicts of interest which gives the audience a good idea about the magnitude of vested financial interests.

Third, we need to pressure advocates of particular options for handling CRISPR/Cas to give a precise rationale for their favored policies in a more or less standardized fashion. This is a more demanding requirement: We should ask scientists involved in debates on the ethical issues with the CRISPR/Cas technology to write and sign a mission statement and upload this mission statement in the registry mentioned before. Such a statement could include answers to a series of questions, which relate to the development of CRISPR/Cas policies:

1. *Organizational feature of public and semi-public debates on ethical issues of the CRISPR/Cas technology*: What organizational model for public and semi-public debates do you prefer for what reasons? What is your role in public and semi-public debates? Who should have access to debates on ethical issues of the CRISPR/Cas technology? Should others defer to your moral assessment? etc.
2. *Responsible pathway to clinical applications*: Do you advocate for a responsible pathway to clinical applications with the aim of heritable human genome editing, for treatment, risk reduction or enhancement? Do you advocate for a responsible pathway to clinical applications with the aim of somatic human genome editing, for treatment, risk reduction or enhancement? etc.
3. *Moratorium on human genome editing*: In case you agree that we should implement a moratorium: What is the scope of the moratorium? What is your justification for a moratorium and how do the arguments for a moratorium outweigh arguments in favor of research freedom? How should we implement a moratorium? In case you disagree that we should implement a moratorium: Why do potential risks not override the justification for a moratorium? How should we, alternatively, prohibit misapplications of CRISPR/Cas in various scenarios? etc.
4. *Moral framework based in your thinking about (1), (2) and (3)*: What are your reasons for adopting specific moral frameworks relating to the usage of non-human animals in basic research, the usage of human embryos and human embryonic stem cells, the selection of target diseases? etc.
5. *Conflicts of interest and conflicts of commitments*: Given your answers to questions in (1) to (3): how would the respective measures affect your financial situation or affiliation to commercial entities? etc.

The three measures proposed here aim at increasing the transparency of public and semi-public debates by requiring detailed disclosure of conflicts of interest and conflicts of commitment. Yet, reviewing the arguments against reliance on the moral expertise of scientists, the argument from a lack of political mandate as well as the list of ploys that might be used to influence public debates (see section Experts in moral debates on the ethical issues of CRISPR/Cas technology and policy making), we should see transparency as only the very first step toward securing a better discourse setting. In the context of this contribution, I can only gesture at some strategies which go beyond the mere minimal requirement of transparency. Based on the recent literature on science communication (Davies and Horst, 2016; Medvecky and Leach, 2019), there are at least two further recommendations which might supplement improved transparency requirements. The first is to put a stronger focus on the ethics of science communication (Medvecky and Leach, 2019). The second is to work toward a diversification of formats for science communication and dialogues between multiple stakeholders (Riise, 2012). The ethics of science communication should be included in curricula in postgraduate education, e.g., research ethics and scientific publication ethics courses. Here the didactic aim should be to make clear that integrity of the communication of science is a condition for a constructive relationship between science and society and for functional policy making.

A diversification of formats for science communication is important to come closer to the ideal of an ethical debate between multiple stakeholders and activists. Alternative types of venues should be created to increase the likelihood of citizens and activists actually engaging in open debates about the ethical issues of CRISPR/Cas. These types of venues could include science cafés, student or science parliaments, student or pupil forums, junior science cafés, citizens' conferences, consensus conferences, citizens' exhibitions, twenty-first century town meetings and joint fact finding (Riise, 2012). In addition to this, one core principle in organizing these venues for debating ethical issues should be to withhold the right to select and invite representatives for the various groups of stakeholders from experts working in CRISPR/Cas technology who have conflicts of interest with respect to the issues discussed. Many universities and research institutions have established offices for science communication and citizen science who could handle the organization, so that a clear separation between the invitation of interest groups and scientific responsibilities—like review of submissions, selection of keynote speakers—is guaranteed.

## Conclusion

The main thesis of this contribution was that we should establish stricter and more comprehensive requirements regarding the disclosure of conflicts of interest and conflicts of commitments in the context of debates on CRISPR/Cas-based human genome editing and change our stance toward the idea that scientific experts can naturally be treated as moral experts.

The promises and prospects of the CRISPR/Cas technology for scientific progress and economic prosperity set strong incentives to disregard established principles of good scientific practice, codes of conduct from bioethics and research protocols. These codes have been established to safeguard the epistemic and moral integrity of research and publications processes as well as protecting society and the environment. The case of He Jiankui illustrates both a failure of science to effectively anticipate the dangers of the new CRISPR/Cas technology and the necessity for an organized attempt to establish boundaries on an international and national level. Two current debates on CRISPR/Cas that can be seen as directly motivated by the case of He concern a moratorium on specific types of genome editing (in particular heritable human genome editing as well as genetic enhancement) and the conditions of a responsible pathway to clinical applications. Within this context, this paper indicated serious potential problems resulting from the presence of conflicts of interest in CRISPR/Cas policy making.

Three measures were proposed to address these problems: a registry for conflicts of interest of scientists, a change in our attitude toward leading experts on the CRISPR/Cas technology in the context of science advocacy, and a mission statement for scientists engaged in public advocacy for CRISPR/Cas policies. The latter would foster our ability to evaluate certain positions in the debates about a moratorium and a so-called responsible pathway toward human germline editing. In addition to these measures to increase transparency in public and semi-public debates on the ethical implications of the CRISPR/Cas technology, I also indicated the need to promote ethical science communication as a topic in postgraduate education as well as the diversification of venues for science communication and the separation of the invitation of interest groups and scientific responsibilities to set the stage for public debates.

Throughout this contribution I tried to make a case for a more cautious stance with regard to conflicts of interests and gave some reasons to believe that conflicts of commitment, i.e., conflicts between a set of primary interest resulting from the adoption of different professional roles, could be a serious issue in policy making processes relating to heritable genome editing. Yet, it is plausible to assume that the mechanisms described in section A plea for caution constitute a more general issue, which is similar to what James Kidd described in a series of publications as “epistemic corruption” (Kidd, 2015, 2019, 2020; Biddle et al., 2017, p. 172–173). Kidd's version of the concept of epistemic corruption describes the phenomenon that “[…] damage [is] done to people's epistemic character by their subjection to conditions or processes that erode epistemic virtues such as curiosity and thoughtfulness and facilitate the epistemic vices like dogmatism or closedmindedness” (Kidd et al., 2021, p. 152). Kidd primarily focusses on epistemic corruption in academic education and is generally concerned with a loss of epistemic virtues in professional agents. What I describe as conflicts of commitment in policy making, which take the form of biased decision making in moral deliberation or the participation in moral deliberation as an (ideally) impartial informant, could count as a corruption of moral virtues due to the presence of epistemic interests. I am concerned that something like this could exist in ethical debates on the limits of biomedical research—e.g., in debates on the morality of animal experimentation, genome editing, human stem cells (etc.). For instance, if a scientist depends on the usage of human embryonic stem cells in her research, she might lean in favor, since she has epistemic interests conducting research with stem cells. Likewise in CRISPR/Cas research, experts might favor a responsible pathway, since their epistemic preferences are not compatible with a moratorium on basic research, thus they adjust their moral framework and advocate for moral guidelines, which create sufficient space for their research.^23^ One reason for such a pattern of thinking might be a commonly found purely epistemic axiology of science (“axiology” means a theory of aims for a research field), which defines the aim of research in purely epistemic terms, e.g. finding empirical adequate theories or figuring out a technical solution for a certain problem (etc.). Adopting a restrictive stance regarding basic research then seems hardly justifiable or even necessary anymore. Also one could make a case, that—due to such a purely epistemic axiology—epistemic interest would prima facie count as primary interest. Yet, if you adopt a mixed axiology, according to which the aim of research consist, for instance, in finding research knowledge which is socially valuable and attained with morally acceptable means, then you could make a case that social utility of research topics and moral acceptability of research methods is routinely in conflict with epistemic preferences. Thus, it constitutes a genuine case of a conflict of commitment between epistemic preferences which dominate your professional roles as a seeker of scientific knowledge, e.g., in a laboratory, and your moral obligations as someone who participates in scientific self-regulation by developing research policies.

## Data Availability Statement

The original contributions presented in the study are included in the article/supplementary material, further inquiries can be directed to the corresponding author.

## Author Contributions

The author confirms being the sole contributor of this work and has approved it for publication.

## Conflict of Interest

The author declares that the research was conducted in the absence of any commercial or financial relationships that could be construed as a potential conflict of interest.

## Publisher's Note

All claims expressed in this article are solely those of the authors and do not necessarily represent those of their affiliated organizations, or those of the publisher, the editors and the reviewers. Any product that may be evaluated in this article, or claim that may be made by its manufacturer, is not guaranteed or endorsed by the publisher.

## Footnotes

^1^Chinese family names are written before the first name, thus He is the family name and Jiankui the first name.

^2^Although the current debate on a moratorium on human germline editing is related to CRISPR/Cas, other types of genome editing technologies would be affected by such a moratorium as well, these include engineered nucleases like meganucleases, Zinc finger nucleases (ZFN), transcription activator-like effector nucleases (TALENs) and Nucleobase Modification (BASE Editing). I would like to thank one reviewer for mentioning this point.

^3^I will speak of “*the* CRISPR/Cas technology” (definite description for the sum of basic knowledge on CRISPR/Cas mechanisms and technical applications), while one might also conceive of it as rather a series of technologies derived from CRISPR/Cas immune systems in bacteria and archaea.

^4^For more details on these and other challenges, see Chan and Sternberg (2019). For a comparative overview on the various reports and ethics statements of ethics commissions, see Brokowski (2018), for an overview on policies on human germline editing, see Baylis et al. (2020). I refer to Carolyn Brokowski's meticulous work instead of adding all the references individually.

^5^https://www.nih.gov/about-nih/who-we-are/nih-director/statements/statement-claim-first-gene-edited-babies-chinese-researcher (retrieved August 31, 2021).

^6^This program was criticized by the US National Institutes of Health (NIH) for taking unfair advantage of US research efforts by hiring scientists with Chinese heritage who had been trained in the US. It was also criticized for a lack of transparency with regard to commercial relationships between US research and Chinese business ventures and some suspected that it was an attempt to conduct research espionage (Hvistendahl, 2014; Cohen and Malakoff, 2019; Mervis, 2019, 2020a,b; Staff, 2020).

^7^The company's website is currently offline, a snapshot can be found on archive.org. https://web.archive.org/web/20181228100437; http://www.directgenomics.com/ (retrieved March 10, 2022).

^8^Helicos went bankrupt in 2012 and SeqLL bought all its intellectual property and hardware. http://seqll.com (retrieved September 1, 2021).

^9^The entire talk as well as the discussion are available on youtube.com. https://www.youtube.com/watch?v=tLZufCrjrN0 (retrieved September 3, 2021).

^10^http://www.xinhuanet.com/english/2019-12/30/c_138666892.htm (retrieved December 17, 2021).

^11^https://www.youtube.com/watch?v=th0vnOmFltc, Retrieved 01-09-2021. One of the first science journalists reporting on the case was Antonio Regalado (MIT Technology Review), who published an investigative article on the same day. https://www.technologyreview.com/2018/11/25/138962/exclusive-chinese-scientists-are-creating-crispr-babies/ (retrieved September 1, 2021).

^12^Also, in a study published in 2019 (Wei and Nielsen, 2019a), which was later retracted due to bias in the underlying data of the UK Biobank (Callaway, 2019; Wei and Nielsen, 2019a,b; Maier et al., 2020), critics worried that a homozygous CCR5-Δ32 mutation is associated with an increased mortality.

^13^https://www.youtube.com/watch?v=aezxaOn0efE (retrieved September 3, 2021).

^14^Also, the WHO proposed a global registry for human research with CRISPR/Cas (Cohen, 2019b). This proposal was recently adopted, the upcoming registry will be a part of the Clinical Trials Registry Platform (ICTRP), which can be accessed under https://trialsearch.who.int (retrieved September 1, 2021).

^15^https://royalsociety.org/-/media/events/2022/03/2022-human-genome-editing-summit/summit-agenda.pdf?la=en-GB&hash=CB1180F8AB4C942433E8DBE1463B9B1E (retrieved December 17, 2021).

^16^See the following systematic reviews on the corrupting influence of financial conflicts of interest on medical research (Bes-Rastrollo et al., 2013; Feuerstein et al., 2013; Lieb et al., 2016; Mandrioli et al., 2016; Probst et al., 2016; Narain et al., 2017; Zhang et al., 2018; Guntin et al., 2019; Hansen et al., 2019; Hendlin et al., 2019; Crow et al., 2022).

^17^This case is in particular deplorable since it fosters stereotypical “wild East” allegations.

^18^https://www.emmanuelle-charpentier-pr.org/ (retrieved December 17, 2021).

^19^https://mcgovern.mit.edu/profile/feng-zhang/ (retrieved December 17, 2021).

^20^As an example: https://www.youtube.com/watch?v=gC_x2XKJjQo (retrieved January 9, 2021).

^21^For instance, Doudna recently (in 2021) gave the Schrödinger Lecture at the Imperial College London (https://www.imperial.ac.uk/news/215993/nobel-laureate-discusses-science-ethics-genome/, retrieved October 3, 2022) and spoke about ethical implications as well as the societal discourse with regard to CRISPR. Also, in an interview with The Harvard Gazette she considered herself as a relative novice in the field of ethics (https://news.harvard.edu/gazette/story/2018/05/crispr-pioneer-jennifer-doudna-explains-gene-editing-technology-in-prather-lectures/, retrieved October 3, 2022).

^22^https://www.chronicle.com/article/many-public-universities-refuse-to-reveal-professors-conflicts-of-interest/ (retrieved January 9, 2021).

^23^Just an illustrative comparison, those scientists affected by conflicts of commitment in policy making are like individuals who adopt a pescatarian diet since they have a culinary preference to eat fish, although they would, in principle, agree to a vegan diet if pressured to give good moral reason for their dietary choices.
